# Simple Method for De Novo Structural Determination of Underivatised Glucose Oligosaccharides

**DOI:** 10.1038/s41598-018-23903-4

**Published:** 2018-04-03

**Authors:** Hsu Chen Hsu, Chia Yen Liew, Shih-Pei Huang, Shang-Ting Tsai, Chi-Kung Ni

**Affiliations:** 1 Institute of Atomic and Molecular Sciences, Academia Sinica, P. O. Box 23-166, Taipei, 10617 Taiwan; 2 Department of Chemistry, National Taiwan Normal University, Taipei, 11677 Taiwan; 3 Department of Chemistry, National Tsing Hua University, Hsinchu, 30013 Taiwan

## Abstract

Carbohydrates have various functions in biological systems. However, the structural analysis of carbohydrates remains challenging. Most of the commonly used methods involve derivatization of carbohydrates or can only identify part of the structure. Here, we report a de novo method for completely structural identification of underivatised oligosaccharides. This method, which can provide assignments of linkages, anomeric configurations, and branch locations, entails low-energy collision-induced dissociation (CID) of sodium ion adducts that enable the cleavage of selective chemical bonds, a logical procedure to identify structurally decisive fragment ions for subsequent CID, and the specially prepared disaccharide CID spectrum databases. This method was first applied to determine the structures of four underivatised glucose oligosaccharides. Then, high-performance liquid chromatography and a mass spectrometer with a built-in logical procedure were established to demonstrate the capability of the *in situ* CID spectrum measurement and structural determination of the oligosaccharides in chromatogram. This consolidation provides a simple, rapid, sensitive method for the structural determination of glucose oligosaccharides, and applications to oligosaccharides containing hexoses other than glucose can be made provided the corresponding disaccharide databases are available.

## Introduction

Carbohydrates (or saccharides) are the most abundant biological compounds on Earth^1^. They play crucial roles in many biological processes, such as immune responses, molecular recognition, signalling, and cellular communication^2^. To elucidate the chemical and biological properties of carbohydrates, relevant structure–function relationships must be studied. However, the identification of carbohydrate structure remains difficult^3^ because of the presence of a high number of carbohydrate isomers in a given chemical formula. For example, an oligosaccharide containing six hexoses has more than 10^12^ isomers^4^, and the differentiation of such a high number of isomers by using a single simple analytical method is difficult.

The most frequently used carbohydrate analysis techniques are liquid chromatography (LC)^5^, capillary electrophoresis (CE)^6,7^, nuclear magnetic resonance spectroscopy (NMR)^8^, and mass spectrometry (MS)^9^. The structures of carbohydrates cannot be determined directly by LC and CE. They are determined by the comparison of chromatography to that of databases which the structure of carbohydrates are characterized by other methods, e.g., NMR and MS. NMR and MS are widely applied in the structural analysis of molecules. However, MS requires much less sample than that of NMR, that makes MS particularly useful for the structural analysis of limited amount of available samples, e.g., the carbohydrates extracted from biological systems. Although MS is widely used in the protein structural determination, application of MS to the identification of carbohydrate structures remain challenging due to the low ionisation efficiency of carbohydrates in a mass spectrometer^10–12^, the large amount of carbohydrate isomers, and the similarity of mass spectra between isomers.

Collision-induced dissociation (CID) tandem MS is one of the major methods of determining the structure of carbohydrates^9,13,14^. Several oligosaccharide fragment databases^15–17^ and empirical fragmentation patterns of carbohydrate cations^18–20^, anions^21–26^, and derivatised carbohydrates^13,27–31^ have been used for structural determination. However, these patterns can determine only a part of the structure or are mainly applied to the well-characterised disaccharides and oligosaccharides in databases.

Several advancements in the de novo structural identification of monosaccharides and oligosaccharides have been demonstrated recently. Nagy *et al*. reported a fixed-ligand kinetic method for the determination of monosaccharide absolute configuration^32^. However, this method was not used for the determination of the linkage positions, anomeric configurations, sequences, and branch locations of oligosaccharides. Konda *et al*. reported that the CID of anion m/z 163 exhibited distinct fragmentation fingerprints corresponding to linkage positions. They applied the CID of anion m/z 163 to the linkage determination of ^18^O-labelled linear oligosaccharides^33^. Bendiak *et al*. demonstrated that anion m/z 221 can be used to identify the stereochemistry and anomeric configuration of hexose in oligosaccharides^34–36^. This method has the following limitations: The reducing end must be derivatised, resulting in the structure of two hexoses on the reducing side cannot be determined, anion intensities are usually low that it may take several hours to obtain a mass spectrum with a good signal-to-noise ratio, a complicated mass spectrometer is required, and this method is currently used for only linear oligosaccharides.

Recently we have proposed a new de novo method for determining the entire structure of underivatised oligosaccharides through CID tandem MS of sodium ion adducts^37^. In this study, the structural determination of glucose trisaccharides and tetrasaccharides was demonstrated. This method can be extended to larger oligosaccharides and oligosaccharides containing hexoses other than glucose.

## The approach of de novo structural determination

The new method involves the sequential low-energy resonance excitation CID experiments of sodiated oligosaccharides in a typical ion trap mass spectrometer. However, the sequence of the tandem CID experiment is specially designed according to the dissociation mechanism we found recently^38,39^, and the measured CID spectra are compared with our specially prepared database. In the following sections, we first describe the dissociation mechanism, followed by a description of the database and the scheme of the procedure to determine the structures of oligosaccharides.

### Dissociation mechanism

#### Dehydration and cross-ring dissociation mainly occur on the reducing side of sodiated oligosaccharides

Recent high-level quantum chemistry calculations^12,38,39^ have indicated that glycosidic bond cleavage, cross-ring dissociation and dehydration reactions on the reducing sugar are dissociation channels with low barrier heights for sodiated carbohydrates. By contrast, cross-ring dissociation and dehydration reactions on the nonreducing sugar are dissociation channels with high barrier heights. Coincidently, the sodiation energy of carbohydrates is near or just slightly higher than the dissociation barrier heights of cross-ring dissociation and dehydration reactions on the reducing sugar.

A mass spectrometer with low-energy CID and resonance excitation is used in this method. We selected sodium ion adducts because sodium ions are an efficient dissociation channel discriminator in CID because of their appropriate sodiation energy and the loose transition state property of desodiation. In the process of low-energy CID using resonance excitation in an ion trap, only the parent ions in an ion trap are excited by the dipolar frequency corresponding to the parent ion motion frequency. They accumulate internal energy from collisions with neutral gases. In each collision, only a small amount of translational energy is transferred to the vibrational energy of trapped ions. It requires many collisions for a trapped ion to obtain enough energy and undergo dissociation. Because the amount of energy transferred in each collision is not fixed, the internal energy distribution of parent ions becomes very broad after many collisions. When the accumulated internal energy is larger than the dissociation threshold, dissociation occurs. Dissociation mainly occurs on the channels with low barriers because the energy transfer is slow. However, if the internal energy is large, sodium cations are eliminated before the occurrence of reactions with large dissociation barrier heights^31^ (e.g., cross-ring dissociation and dehydration reactions on the nonreducing sugar). The product ions are trapped in the ion trap, but they cannot be excited by the parent ion motion frequency. Most of the product ions do not have enough excess of internal energy to undergo consecutive cleavages, thus fragment ions of the secondary dissociation are minimized. The combination of low-energy CID, resonance excitation, and sodium adducts guarantees the occurrence of most cross-ring dissociation and dehydration reactions on the reducing sugar.

#### Linkages of the reducing sugar can be determined from the fragmentation patterns of dehydration and cross-ring dissociation

The fragmentation patterns of dehydration and cross-ring dissociation found in sodiated and lithiated disaccharides in previous studies^40–43^ can be extended based on the dehydration mechanism^38^ and the retro-Aldol reaction^38,40^. These fragmentation patterns can be used for linkage determination of the reducing sugar of oligosaccharides. For linear oligosaccharides, these patterns include ^0,2^A_n_ for 1→4 and 1→6 linkages; ^0,3^A_n_ (or ^0,3^X_0_) for 1→3 and 1→−6 linkages; ^0,4^A_n_ for a 1→6 linkage; and dehydration for 1→3, 1→4, and 1→6 linkages. When a branch is located on the reducing sugar, dehydration occurs for (1→6, 1→4), (1→6, 1→3), and (1→4, 1→3) linkages, and cross-ring dissociation on the reducing sugar follows the retro-Aldol reaction; that is, ^0,2^A_n_ (or ^0,2^X_0_) for (1→6, 1→4), (1→6, 1→2), and (1→4, 1→2) linkages and ^0,3^A_n_ (or ^0,3^X_0_) for (1→6, 1→3) and (1→3, 1→2) linkages. The details of the fragmentation patterns of trisaccharides are listed in Supplementary Table S8. In general, different linkages result in different fragmentation patterns, except for a linear oligosaccharide with a (1→4) linkage and a branched oligosaccharide with (1→6, 1→4) linkages. Oligosaccharides with these two linkages can be differentiated using subsequent CID spectra.

#### Disaccharides from the nonreducing side can be generated from the products of dehydration and cross-ring dissociation

Part of the structural determination process includes the dissociation of oligosaccharides into disaccharides by CID in a mass spectrometer; these disaccharides are then *in situ* fragmented into their corresponding fingerprint fragments. To simplify structural determination, a disaccharide is generated only from one side (the reducing or nonreducing side) of parent or fragment ions in each step of CID. Similar concept has been used in negative mode with derivatized oligosaccharide^36^. Derivatization of oligosaccharide on the reducing sugar is crucial in those studies^36^ in order to ensure that the disaccharide fragment is produced only from the nonreducing side of the oligosaccharide during CID. However, the derivatization processes are tedious and time-consuming. Most important, the derivatization changes the structure of the disaccharide on the reducing side. Consequently, the linkage and the anomeric configuration of the disaccharide on the reducing side of oligosaccharide cannot be determined.

To simplify the structural determination process and determine the structure of entire oligosaccharide, we introduce a simple approach to obtain the desired disaccharide fragments from underivatized oligosaccharide unambiguously. We found that generating a disaccharide from only the nonreducing side is easy if dehydration or cross-ring dissociation products are selected as precursor ions. Because dehydration or cross-ring dissociation of sodiation oligosaccharides mainly occurs on the reducing side, the disaccharide generated from parent or fragment ions after dehydration or cross-ring dissociation is mainly the disaccharide from the nonreducing side.

#### Structures of disaccharides are determined according to rules

After the generation of a disaccharide from an oligosaccharide, the structure of the disaccharide can be determined according to the following rules. First, the fragmentation patterns of dehydration and cross-ring dissociation found in sodiated and lithiated disaccharides in previous studies^40–43^ can be directly used in linkage determination. Second, our recent theoretical calculations revealed that dehydration is mainly related to the cis or trans configuration of the O1 and O0 atoms of the reducing sugar^38^. Therefore, the anomeric configurations of the reducing sugar in a disaccharide can be determined using the ratio of dehydration to any dissociation channel that is not related to dehydration. Third, the dissociation mechanism of glycosidic bond cleavage is analogous to that of dehydration; that is, it is related to the cis and trans configuration of the O1 and O0 atoms of the nonreducing sugar of a disaccharide. The anomeric configuration of the glycosidic bond in a disaccharide can be determined using the ratio of glycosidic bond cleavage to any dissociation channel that is not related to glycosidic bond cleavage.

For practical applications, the CID spectra of disaccharides with various linkages and anomeric configurations are measured in advance and prepared as a database. The structures of these disaccharides are determined according to the aforementioned rules. The structures of disaccharides produced from the dissociation of an oligosaccharide are then determined by comparing the measured CID spectra with the database.

### Specially prepared database of disaccharide CID spectra

The α and β anomeric configurations of the sugar at the reducing end typically coexist in a solution. The ratio of these two anomers depends on the solvent, pH, and temperature of the solution. Therefore, the CID spectrum of a given disaccharide depends on sample preparation if these two configurations are not separated before the CID spectrum measurement. Because these two configurations of a given disaccharide were not separated before the CID spectrum measurement in previous studies^40–43^, those CID spectra can only be used for determining linkage positions, not anomeric configurations.

**Figure 1:**
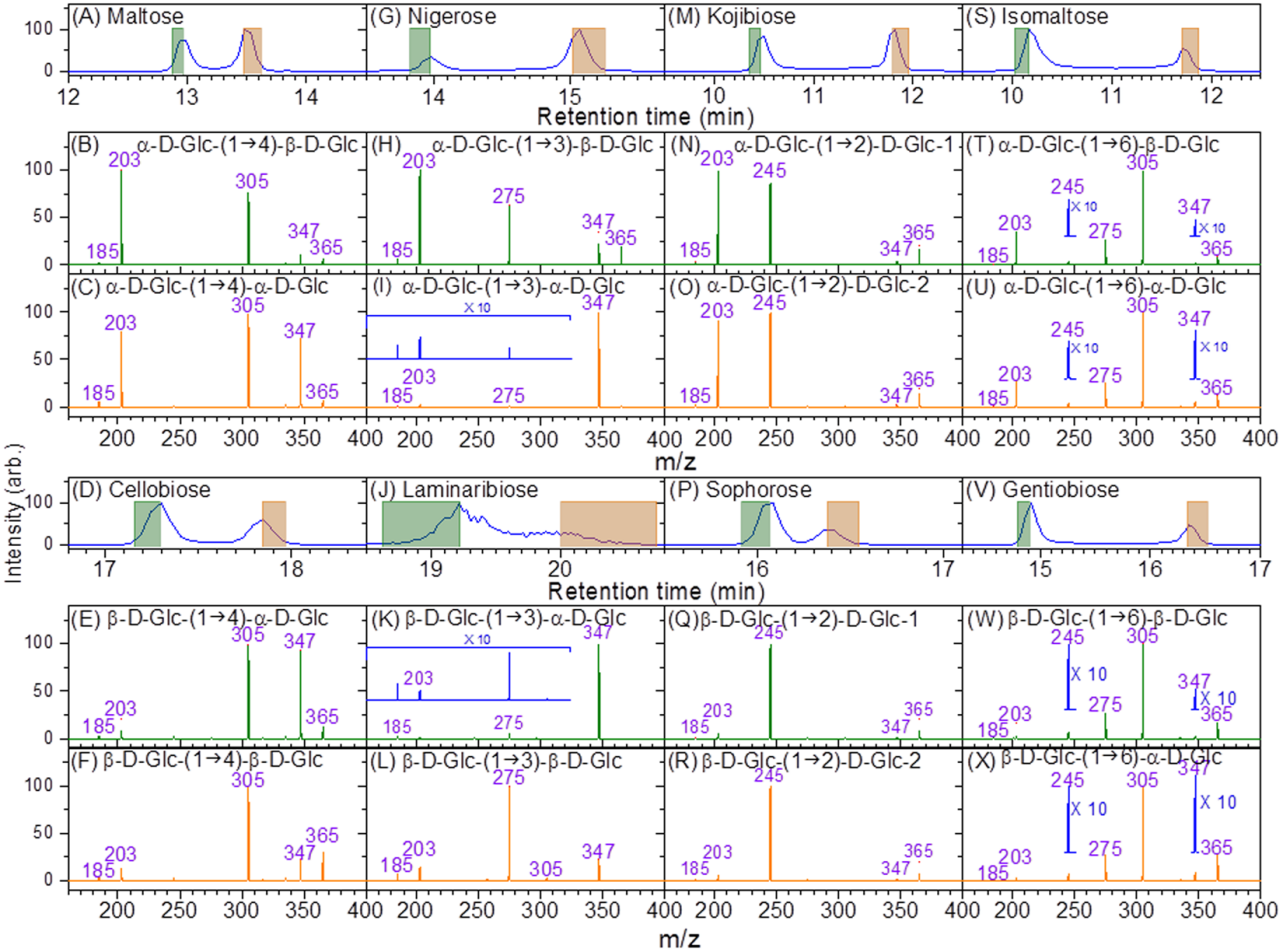
TIC chromatograms and CID spectra of various disaccharides. The green and orange areas in each peak of the chromatogram represent the period over which CID spectra were measured. The corresponding CID spectra are shown in green and orange, respectively.

We constructed our special disaccharide database by separating the two anomeric configurations prior to CID spectrum measurement. Figure 1(A) presents the total ion count (TIC) chromatogram of maltose obtained using the online coupling of high-performance liquid chromatography (HPLC) with electrospray ionisation (ESI) MS (ESI-MS). Two configurations of maltose, namely α-Glc-(1→4)-α-Glc and α-Glc-(1→4)-β-Glc, coexist in a solution. Because mutarotation between configurations is typically slower than separation time in HPLC^44,45^, the separation of these two configurations can be clearly observed on chromatograms. The CID spectra of the two peaks in the chromatogram are presented in Fig. 1(B) and (C), respectively.

A major difference between Fig. 1(B) and (C) is the relative intensity of ion m/z 347, representing the dehydration (elimination of H_2_O) from the reducing end of maltose. This intensity difference of ion m/z 347 can be explained by the dissociation mechanism discovered from high level quantum chemistry calculations^38^. Water elimination mainly occurs through the transfer of the H atom from the O2 atom of the reducing glucose to the O1 atom of the same glucose, followed by C1–O1 bond cleavage. The O1 and O2 of the reducing glucose of α-Glc-(1→4)-α-Glc are in a cis configuration, in contrast to the trans configuration of those of α-Glc-(1→4)-β-Glc. Calculations reveal that the water elimination barrier of the cis configuration is substantially smaller than that of the trans configuration^38^. Therefore, the CID spectrum with a high intensity of ion m/z 347 [Fig. 1(C)], which represents a large branching ratio of the water elimination, is assigned to α-Glc-(1→4)-α-Glc. Similar assignments can be made for the spectra of other disaccharides, except for kojibiose and sophorose, in which the dehydration reaction is a minor channel and mechanism is different.

### Procedures of structural determination

**Figure 2:**
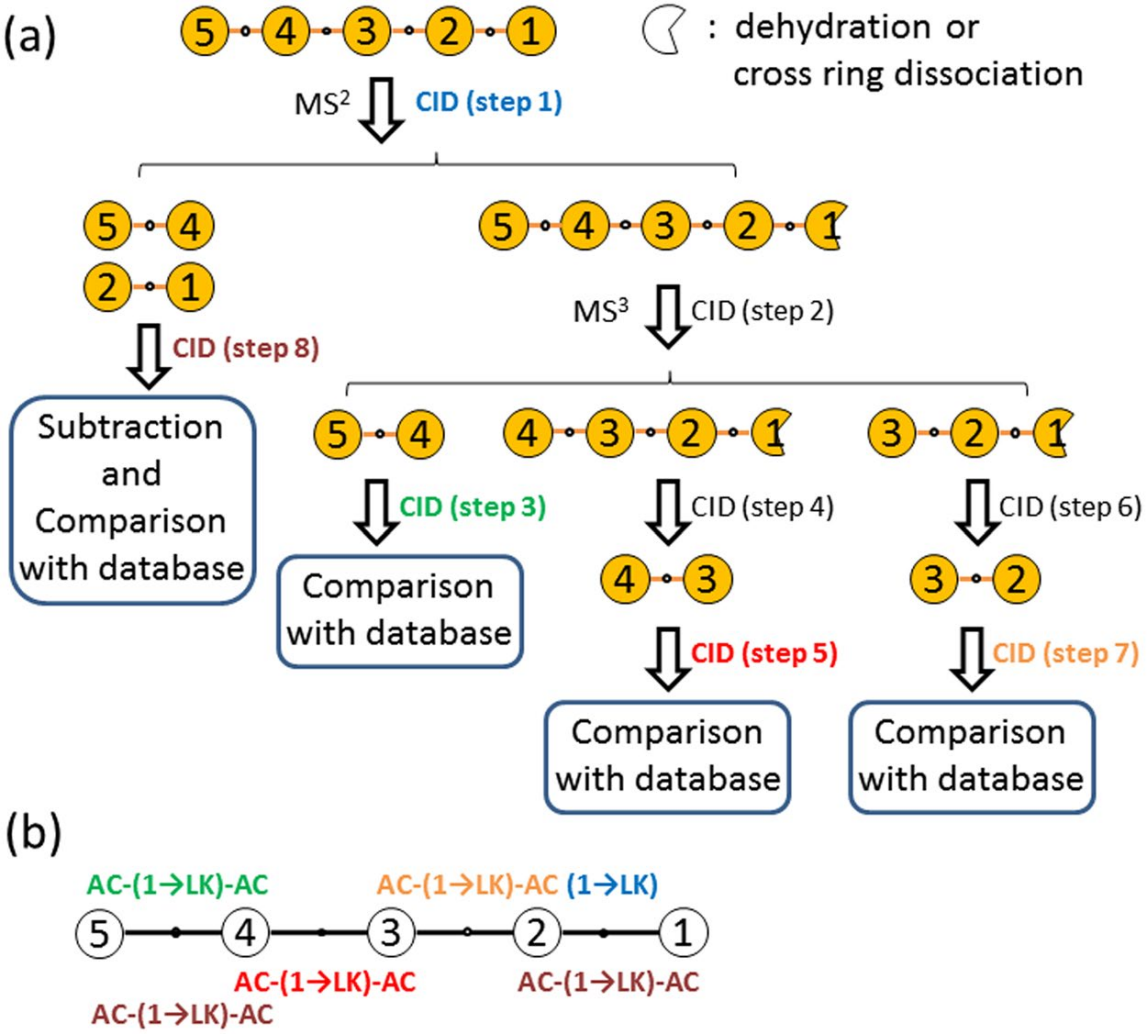
(**a**) Schematic of the procedure for the structural determination of linear pentasaccahrides. The reducing sugar is located at monosaccharide labelled with 1. Only structural decisive fragments are shown. (**b**) Determinations of the anomeric configuration (AC represents α or β) and linkage (LK represents 2, 3, 4, or 6) are colour coded according to the CID steps of the procedure. For example, the anomeric configurations and linkage of the disaccharide comprising monosaccharides 3 and 4, AC-(1-LK)-AC (red), is determined by step 5 (red).

A schematic of the procedure for the structural determination of oligosaccharides is presented in Fig. 2(a); linear pentasaccharides are used as an example. The fragments in Fig. 2(a) are generated according to the aforementioned dissociation mechanism; that is, the cleavage of the glycosidic bond to generate B, C, Y, and Z ions can occur between any two adjoining monosaccharides, but dehydration and cross-ring dissociation only takes place on the reducing sugar. Not all possible fragments are plotted in Fig. 2(a); only structural decisive fragments are shown. The structural determination procedure is as follows. (1) The linkage of the reducing sugar is determined by the aforementioned fragmentation patterns in step 1 (MS2). (2) A disaccharide at the nonreducing side of an oligosaccharide comprising monosaccharides labelled with 4 and 5 is generated from the CID of dehydration or cross-ring dissociation products in step 2. The linkage and anomeric configuration of the glycosidic bond between monosaccharides 4 and 5 and the anomeric configuration of the reducing sugar (monosaccharide 4) are determined in step 3 by matching with the database. Interestingly, the anomeric configuration of the reducing sugar (monosaccharide 4) of a disaccharide comprising monosaccharides 4 and 5 also represents the anomeric configuration of the glycosidic bond between monosaccharides 4 and 3, which can be determined separately from the disaccharide comprising monosaccharides 3 and 4 (in step 5). These two independent approaches provide a crosscheck of the anomeric configuration between monosaccharides 3 and 4. (3) The other disaccharides can be produced by CID from the nonreducing end of various fragment ions, as shown in steps 4 and 6. The linkage and anomeric configurations of corresponding disaccharides can be determined in steps 5 and 7, respectively. (4) Disaccharides from both the reducing (comprising monosaccharides 1 and 2) and nonreducing ends (comprising monosaccharides 4 and 5) of an oligosaccharide are produced in step 1. The CID spectrum of these ions is the sum of these two disaccharide CID spectra. If the structure of one disaccharide is determined, the structure of the other disaccharide can be determined using the CID spectra obtained in step 8 after subtracting the CID spectrum of the disaccharide whose structure is determined. This provides additional information for crosscheck. (5) The structure of the entire oligosaccharide is then determined by the combination of structural information obtained individually from various disaccharides, as illustrated in Fig. 2(b). Most of the anomeric configurations can be determined using more than one approach. For example, the anomeric configuration of monosaccharide unit 4 can be determined by steps 3, 5, and 8. Multiple approaches increase the reliability of this method.

**Figure 3:**
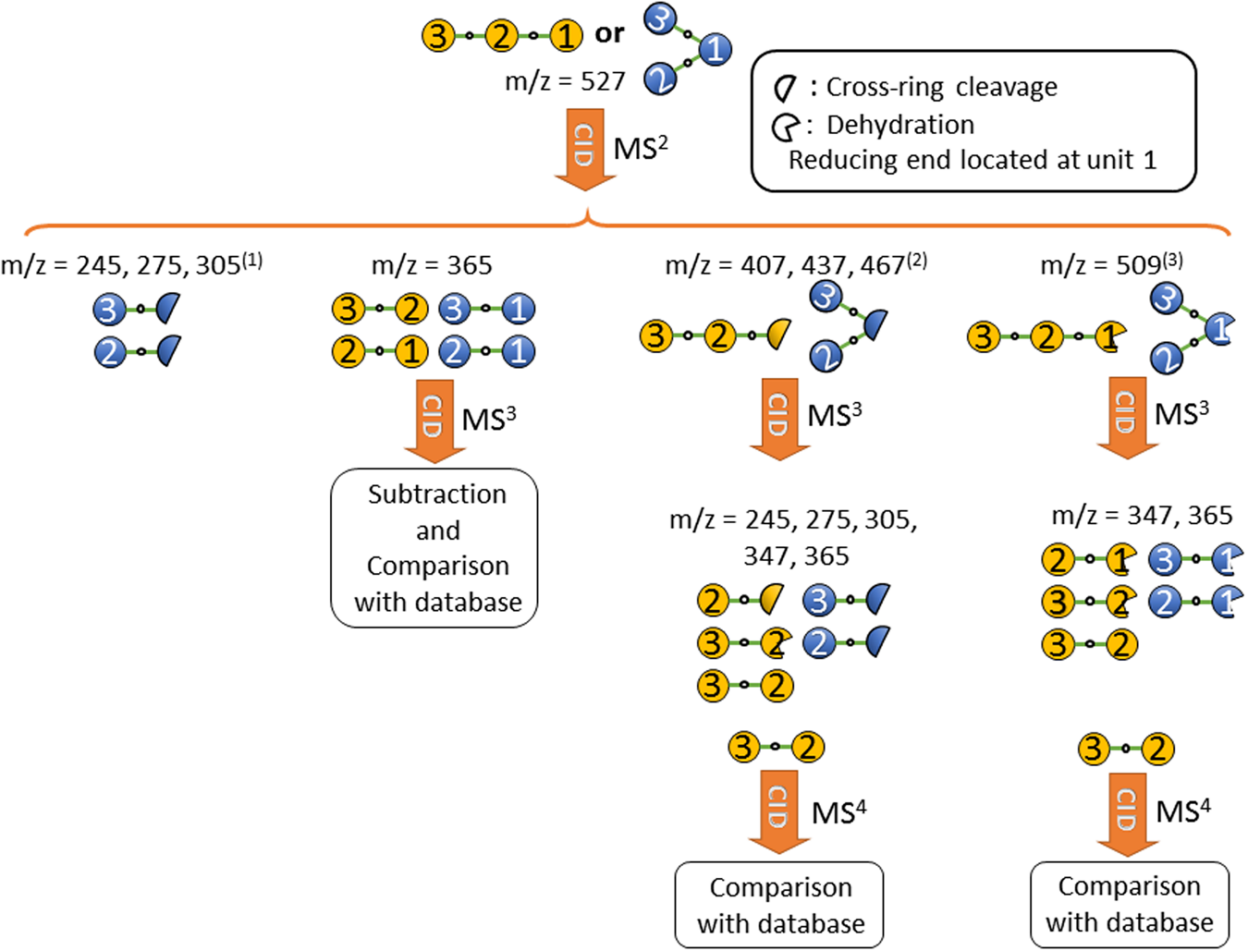
Schematic of the procedure for identifying the structurally decisive ions of trisaccharides and the sequence of CID measurement. (1): Except branched trisaccharides with (1→6, 1→4), (1→4, 1→3), and (1→3, 1→2) linkages. (2): Except linear trisaccharides with a (1→1) linkage on the reducing sugar and branched trisaccharides with (1→6, 1→3), (1→6, 1→2), and (1→4, 1→2) linkages. (3): Except (1→2) or (1→1) linkages on the reducing sugar. The details of the m/z values of fragments are listed in Supplementary Table S8.

A similar procedure can be employed for the structural determination of branched oligosaccharides. The procedure becomes complicated when both linear and branched oligosaccharides are considered. However, once the procedure is established, the sequence of CID spectrum measurement and the structural determination are straightforward. Figure 3 presents a schematic of the procedure for the structural determination of branched and linear trisaccharides. The procedure includes all possible disaccharides that can be generated by CID and the necessary CID spectrum measurement for structural determination. The details of the fragmentation patterns are listed in Supplementary Table S8. The applications of the scheme for structural determination are demonstrated in the next section. A similar scheme for oligosaccharides containing more than three monosaccharides can be developed using the same concept.

## Results and Discussion

### Application for the structural determination of oligosaccharides

#### Panose

**Figure 4:**
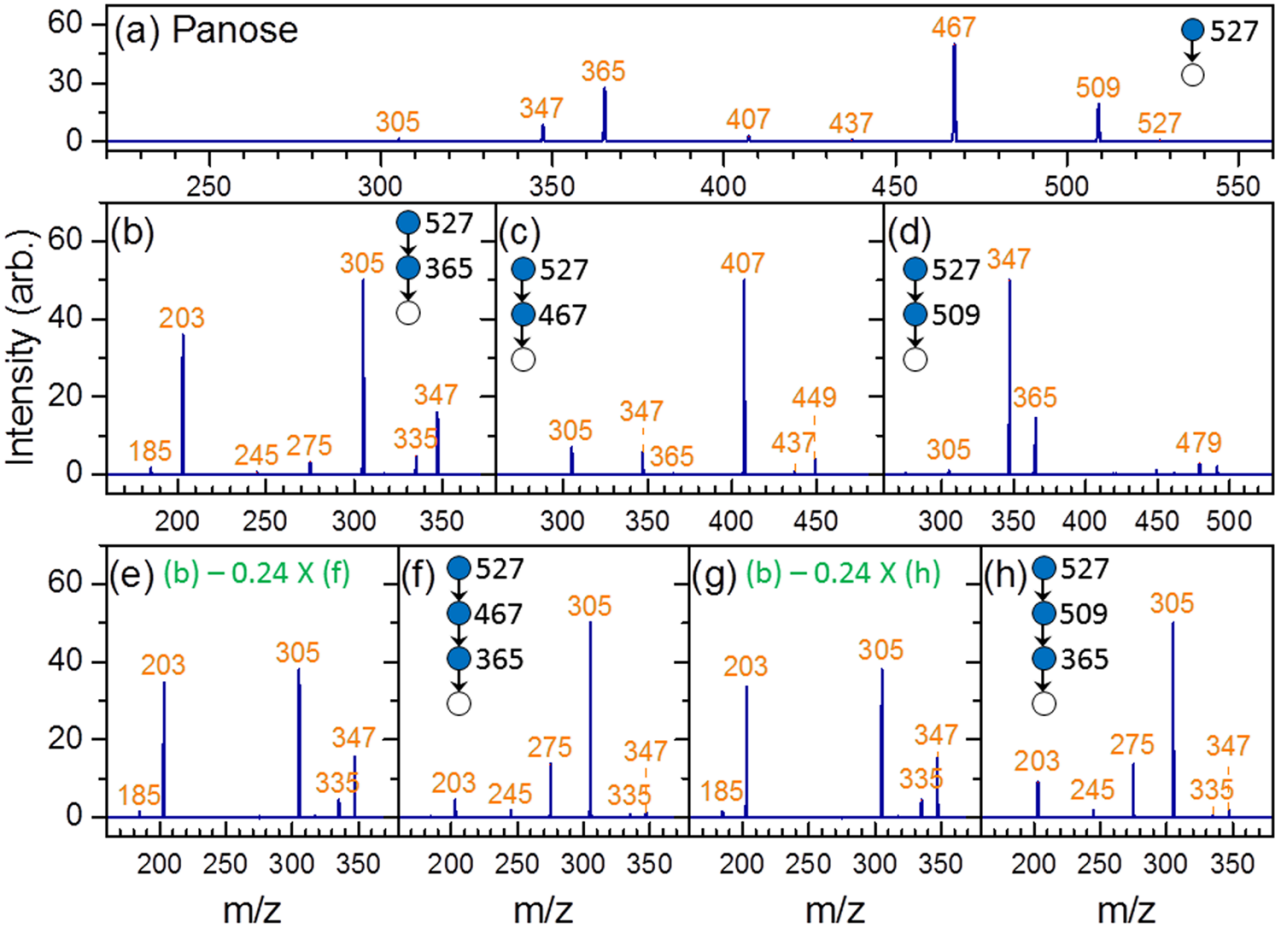
CID spectra of panose. Spectrum in (**e**,**g**) were produced by the subtraction of the spectrum in (**f**,**h**) (weighted by a factor), respectively, from that in (**b**). The weighted factor was chosen such that ion m/z 275 has zero intensity in (**e**,**g**).

The CID spectrum of sodiated panose is displayed in Fig. 4(a). The ions m/z 509 and 467 indicate that the carbohydrate is a linear trisaccharide with a 1→4 linkage or a branched trisaccharide with 1→4 and 1→6 linkages on the sugar of the reducing end according to the fragmentation patterns shown in Supplementary Table S8. Ion m/z 365 in the CID spectrum of 527→509→fragments [Fig. 4(d)] indicates that the carbohydrate is a linear trisaccharide according to the scheme in Fig. 3. The CID spectrum of the disaccharide from the nonreducing side, 527→509(B_3_)→365 (C_2_/B_3_)→fragments, is presented in Fig. 4(h). A comparison of this spectrum with that in Fig. 1 suggests that this disaccharide is α-Glc-(1→6)-α-Glc. Therefore, the trisaccharide is determined to be α-Glc-(1→6)-α-Glc-(1→4)-Glc.

Dehydration is a minor dissociation channel of the disaccharide with a 1→6 linkage. If noise happens to appear near ion m/z 347, it may affect the identification of the anomeric configuration of the reducing sugar. Here, we crosschecked the anomeric configuration of the 1→6 linkage by using a different approach. The sodiated disaccharide ion m/z 365 produced from 527→365 could be the disaccharide on the reducing side (Y_2_ ion) or that on the nonreducing side (C_2_ ion). The CID spectrum of 527→365→fragments [Fig. 4(b)] is the sum of the spectra of these two disaccharides, weighted by the percentage of each disaccharide produced in CID. These two spectra include one spectrum from the disaccharide with a 1→6 linkage [i.e., Fig. 4(h)] and the spectrum from the disaccharide with a 1→4 linkage [i.e., one of Fig. 1(B,C,E or F)]. Subtraction of the spectrum in Fig. 4(h) from that in Fig. 4(b) yields the spectrum in Fig. 4(g), from which the carbohydrate can be unambiguously identified as α-Glc-(1→4)-α-Glc [Fig. 1(B)] or α-Glc-(1→4)-β-Glc [Fig. 1(C)]. Consequently, the structure of this trisaccharide is determined to be α-Glc-(1→6)-α-Glc-(1→4)-Glc.

An alternative approach for structural determination involves 527→467(^0,2^A_3_)→365(C_2_/^0,2^A_3_)→fragments. This approach shares the same first step of the aforementioned method, i.e., the ions m/z 509 and 467 in the CID of 527→fragments [Fig. 4(a)] indicate that the carbohydrate is a linear trisaccharide with a 1→4 linkage or a branched trisaccharide with 1→4 and 1→6 linkages on the reducing sugar according to the fragmentation patterns shown in Supplementary Table S8. The difference of this alternative approach is the use ion m/z 467 instead of m/z 509 from the CID of 527→fragments for subsequent CID. Ion m/z 347 in the CID spectrum of 527→467→fragments, [Fig. 4(c)], indicates that the carbohydrate is a linear trisaccharide with a 1→4 linkage at the reducing sugar according to the scheme in Fig. 3. A comparison of Fig. 1 with the disaccharide CID spectrum produced from 527→467(^0,2^A_3_)→365(C_2_/^0,2^A_3_)→fragments [Fig. 4(f)] suggests that the disaccharide on the non-reducing side is α-Glc-(1→6)-Glc. Subtraction of the CID spectrum in Fig. 4(f) from the CID spectrum in Fig. 4(b) yields the spectrum in Fig. 4(e), from which the carbohydrate can be unambiguously identified as α-Glc-(1→4)-α-Glc [Fig. 1(B)] or α-Glc-(1→4)-β-Glc [Fig. 1(C)]. Consequently, the structure of this trisaccharide can be determined as α-Glc-(1→6)-α-Glc-(1→4)-Glc. The spectrum matching by using calculations of similarity for these three different approaches are presented in Supplementary Table S4.

#### β-Glc-(1→3)-β-Glc-(1→4)-Glc

**Figure 5:**
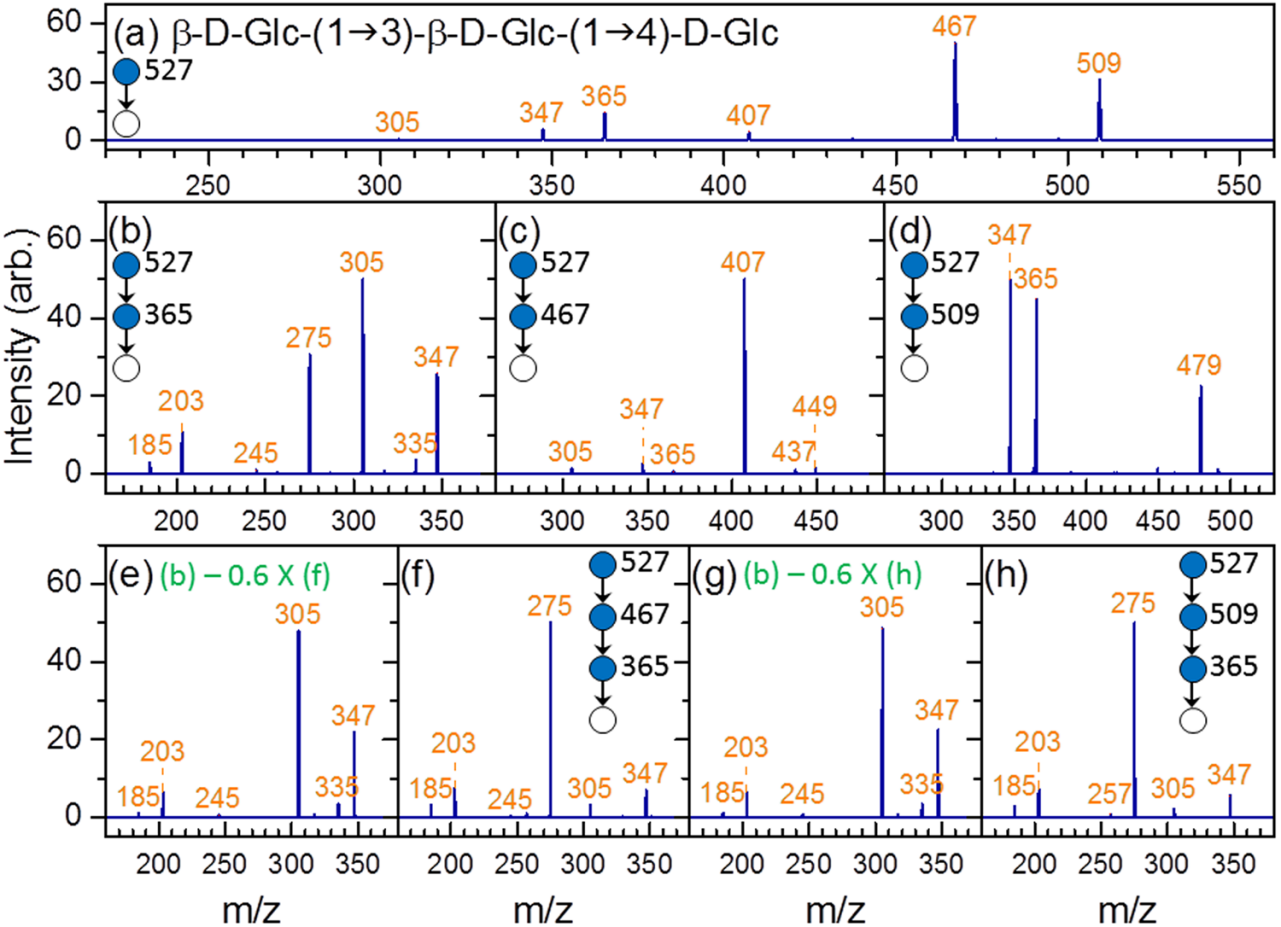
CID spectra of β-Glc-(1→3)-β-Glc-(1→4)-Glc. Spectrum in (**e**,**g**) were produced by the subtraction of the spectrum in (**f**,**h**) (weighted by a factor), respectively, from that in (**b**). The weighted factor was chosen such that ion m/z 275 has zero intensity in (**e**,**g**).

The structural determination procedure for this trisaccharide is similar to that for panose. Ions m/z 509 and 467 in the CID spectrum of ion m/z 527 [Fig. 5(a)] suggest that the carbohydrate is a linear trisaccharide with a 1→4 linkage or a branched trisaccharide with 1→4 and 1→6 linkages on the reducing sugar, and ion m/z 365 in the CID spectrum of 527→509→fragments [Fig. 5(d)] indicates that the trisaccharide is linear according to fragmentation patterns shown in Supplementary Table S8. Comparing Fig. 1 with the CID spectrum of the disaccharide at the nonreducing side, 527→509(B_3_)→365(C_2_/B_3_) →fragments [Fig. 5(h)], suggests that the disaccharide is β-Glc-(1→3)-β-Glc. Therefore, the trisaccharide can be identified as β-Glc-(1→3)-β-Glc-(1→4)-Glc. Analogous to panose, alternative approaches can be used for this trisaccharide to crosscheck the structure. The alternative approaches include using (1) 527→467(^0,2^A_3_) →fragments and 527→467(^0,2^A_3_)→365(C_2_/^0,2^A_3_)→fragments or (2) 527→365→fragments. The CID spectra for the alternative approaches are illustrated in Fig. 5(b,c,e and (f). Both alternative approaches obtain the same results. The spectrum matching by using calculations of similarity is presented in Supplementary Table S5.

#### Isopanose (a branched trisaccharide)

**Figure 6:**
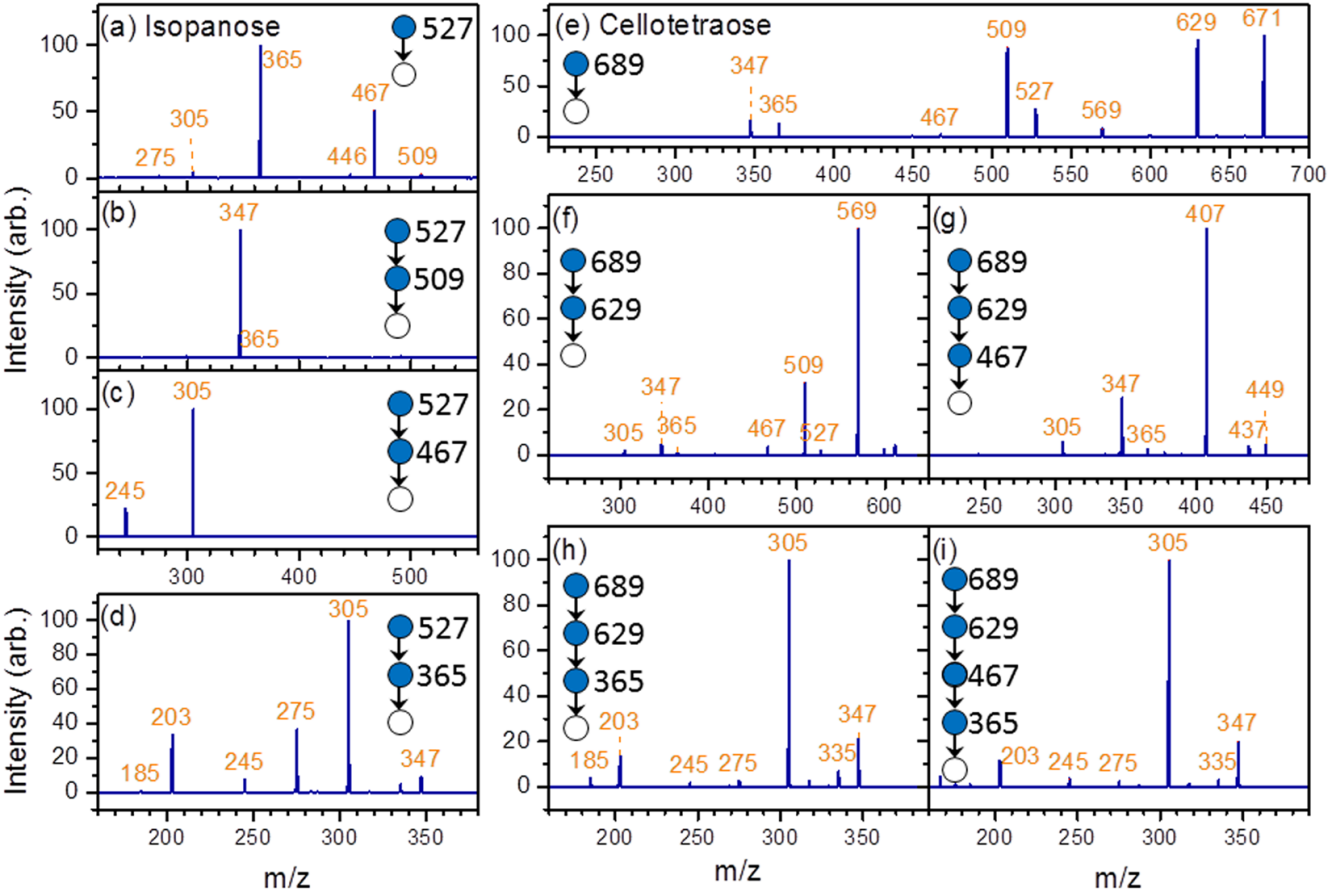
CID spectra of (**a**–**d**) isopanose and (**e**–**i**) cellotetraose.

Branched oligosaccharides have more than one non-anomeric carbons (C2, C3, C4, or C6) of a given monosaccharide connected to another sugar. Most of the present de novo structural determination methods are applied to linear oligosaccharides. Structural identification of branched oligosaccharides remains challenging. The CID spectrum of sodiated isopanose, α-Glc-(1→4)-[α-Glc-(1→6)]-Glc, a branched trisaccharide, is illustrated in Fig. 6(a). The ions m/z 509 and 467 indicate that the carbohydrate is a linear trisaccharide with a 1→4 linkage or a branched trisaccharide with 1→4 and 1→6 linkages, according to the fragmentation patterns shown in Supplementary Table S8. Ions m/z 365 produced from 527→509→fragments [Fig. 6(b)] or ions m/z 347 and 365 produced from 527→467→fragments [Fig. 6(c)] were not observed. According to the scheme in Fig. 3, this finding indicates that the carbohydrate is a branched trisaccharide.

The CID spectrum of 527→365→fragments [Fig. 6(d)] is the sum of the spectra of disaccharides with 1→6 and 1→4 linkages. Subtraction of the spectrum in Fig. 1(W) or (X) [β-Glc-(1→6)-Glc] from that in Fig. 6(d) yields a spectrum of large intensity of ion m/z 203 and near zero intensities of ions m/z 245, 275, and 305, which does not match with any spectrum of disaccharides with 1→4 linkage. By contrast, subtraction of the spectrum in Fig. 1(T) or (U) [α-Glc-(1→6)-Glc] from that in Fig. 6(d) yields a spectrum of near zero intensity for all ions, indicating that the disaccharide produced from 527→365 is mainly α-Glc-(1→6)-Glc and almost no disaccharide with 1→4 linkage is produced. At this moment, we can only determine the linkages of both branches and the anomeric configuration of one branch. The spectrum matching by using calculations of similarity is presented in Supplementary Table S6.

If the quantities of disaccharides with 1→6 and 1→4 linkages produced through CID of parent ions are not very different, the anomeric configurations of both disaccharides can be determined using the method similar to the structural determination procedure of panose. Unluckily, this does not occur in isopanose. However, there is no reason that all branched oligosaccharides break only the glycosidic bond of one branch without breaking that of the other branch. The determination of only one anomeric configuration in isopanose is not some kind of inherited problems of this method when sequencing branched sugars. Unfortunately, isopanose is the only branched glucose-trisaccharide commercially available at this moment. We do not have another branched glucose-trisaccharide available to test our method.

#### Cellotetraose

**Figure 7:**
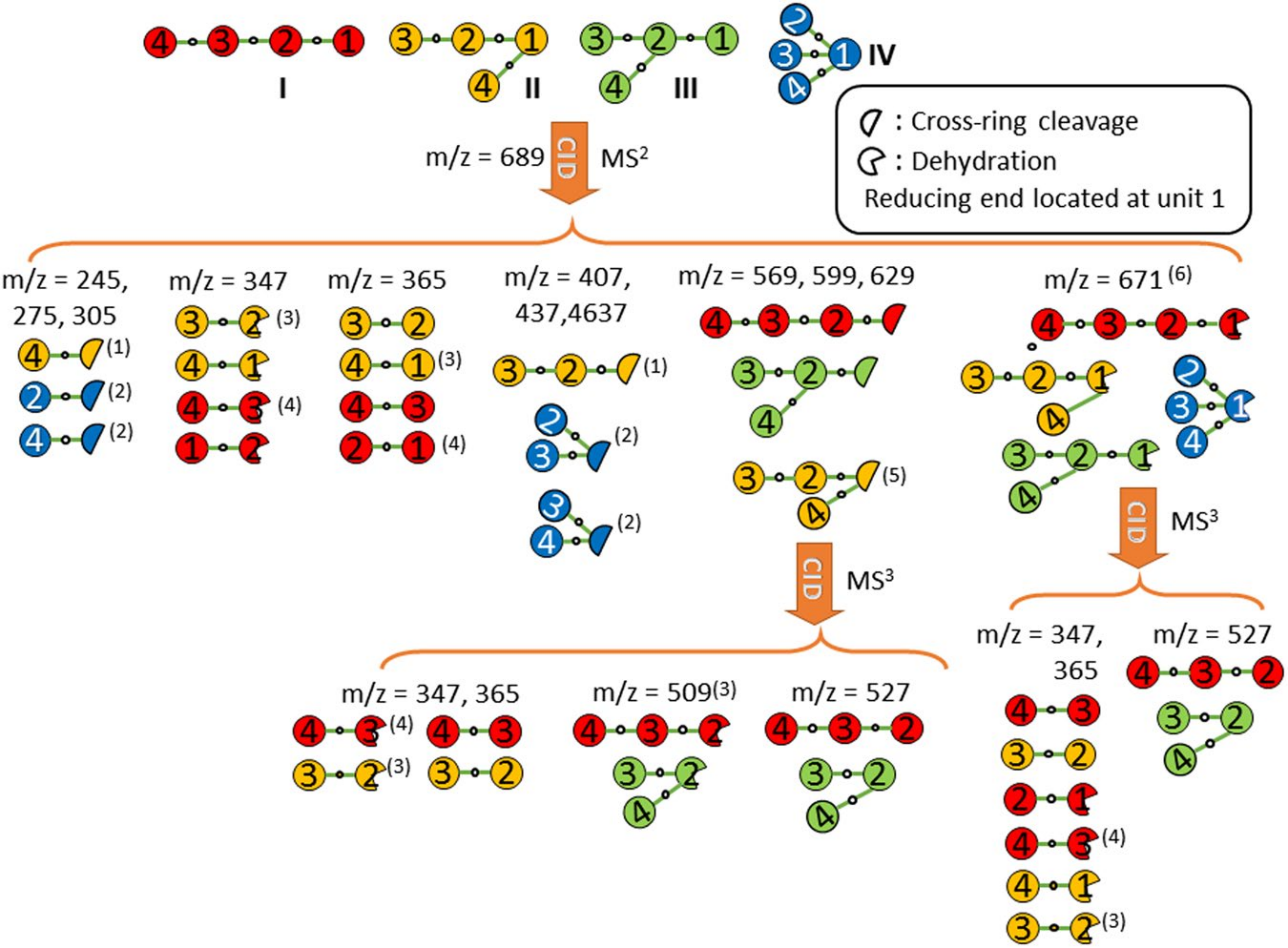
Scheme for identifying the structurally decisive ions of tetrasaccharides. Only fragments related to the determination of the four types of tetrasaccharides are illustrated. Linkage (1→1) is not considered in this scheme. (1): Except (1→6, 1→4), (1→4, 1→3), and (1→3, 1→2) linkages on reducing sugar. (2): Except (1→6, 1→4, 1→3) and (1→4, 1→3, 1→2). (3): Except (1→2) between monosaccharides 2 and 1. (4): Except (1→2) between monosaccharides 3 and 2. (5): Only for (1→6, 1→4) and (1→3, 1→2) on reducing sugar. (6): Without (1→2) on reducing sugar.

There are four types of tetrasaccharides, namely the linear tetrasaccharide (I), branched tetrasaccharide on the reducing sugar (II), branched tetrasaccharide on the nonreducing sugar (III), and tetrasaccharide with two branches on the reducing sugar (IV). The scheme used to differentiate these four types of tetrasaccharides is illustrated in Fig. 7.

Ions m/z 365 and 347 produced through the CID of ions m/z 689 [Fig. 6(e)] and ions m/z 527 and 509 produced through the CID of 689→629→fragments [Fig. 6(f)] suggest that the tetrasaccharide is linear according to the scheme illustrated in Fig. 7. The generation of ions m/z 671(B_4_) and 629(^0,2^A_4_) from the sodiated tetrasaccharide ion m/z 689 [Fig. 6(e)] indicates a 1→4 linkage between the first two monosaccharides on the reducing side. The CID spectrum of 689→629(^0,2^A_4_)→365 (C_2_/^0,2^A_4_)→fragments [Fig. 6(h)] suggests that the structure of the first two monosaccharides on the nonreducing side of the tetrasaccharide is β-Glc-(1→4)-β-Glc, and the CID spectrum of 689→629(^0,2^A_4_)→467(Y_3_/^0,2^A_4_)→365 →fragments [Fig. 6(g) and (i)] suggests that the structure of the two monosaccharides at the centre of the tetrasaccharide is β-Glc-(1→4)-β-Glc. A combination of these spectra can be used to identify the structure as β-Glc-(1→4)-β-Glc-(1→4)-β-Glc-(1→4)-Glc. The spectrum matching by using calculations of similarity is presented in Supplementary Table S7.

### ***In situ*** CID spectrum measurement

Common commercially available mass spectrometers are equipped with sophisticated software to perform experiments automatically. However, if no appropriate guidance is established for the selection of daughter ions for tandem mass spectrum measurement, time and precious samples are wasted because many CID spectra do not provide the structural information necessary for structural determination. The situation becomes critical when the amount of sample is limited, which is common when carbohydrates are extracted from biological samples. By contrast, structurally decisive fragments can be identified according to the schemes shown in Fig. 3. The entire structural determination can be considerably simplified by measuring only the CID spectra of these fragments.

**Figure 8:**
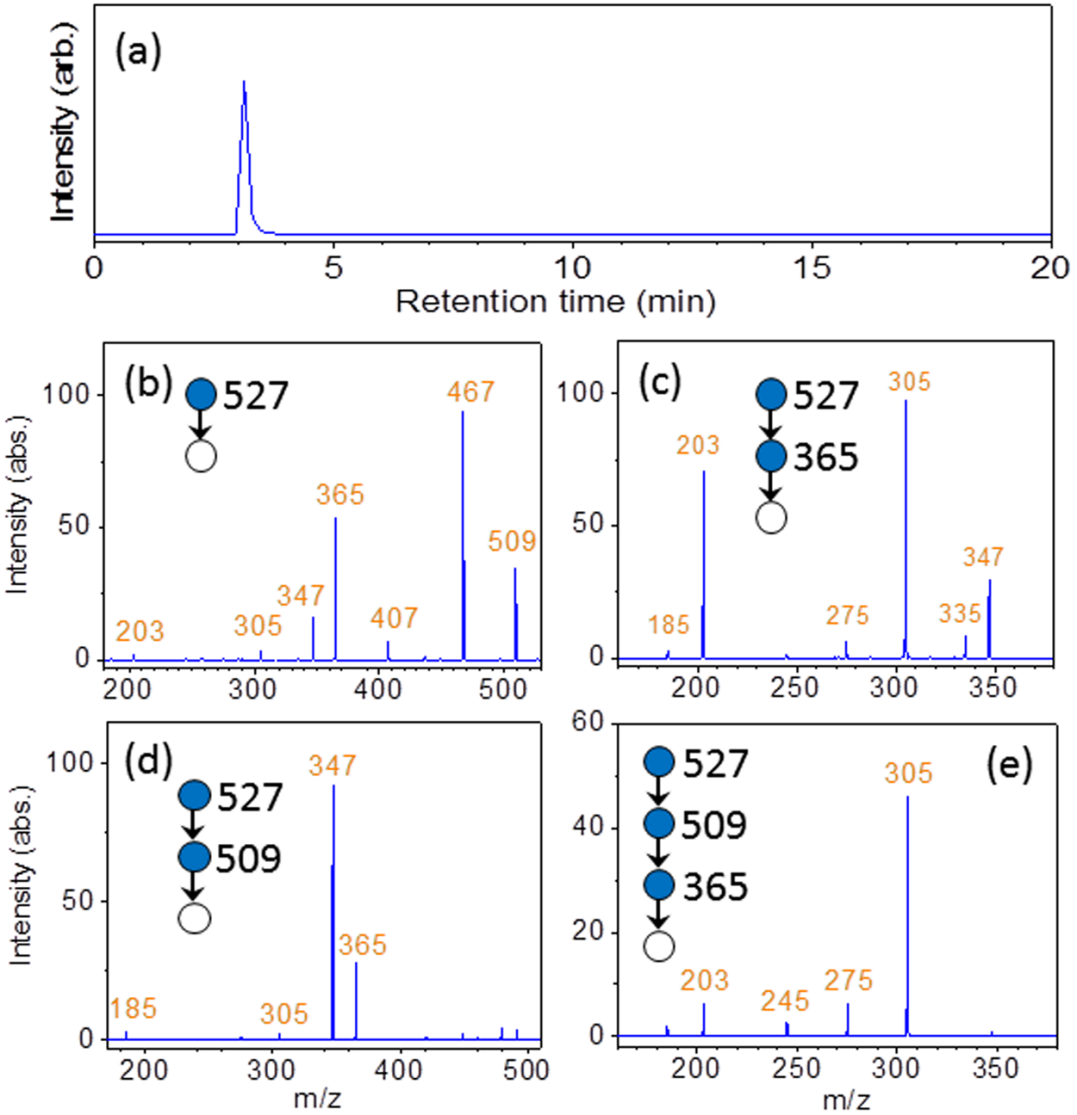
(**a**) TIC chromatogram of panose obtained through the online coupling of HPLC with ESI-MS. (**b**–**e**) CID spectra obtained according to the logical procedures of Fig. 3 during the appearance of the peak in the chromatogram.

A mass spectrometer with built-in logical procedures was established for *in situ* CID spectrum measurement and structural determination. Figure 8(a) shows the TIC chromatogram of a trisaccharide, panose, obtained through the online coupling of HPLC with ESI-MS. While the oligosaccharide was passed through liquid chromatography, the mass spectrometer *in situ* performed all the necessary CID spectrum measurements. Figure 8(b–e) shows the CID spectra obtained during the appearance of a peak in the chromatogram. The results are the same as the CID spectra shown in Fig. 4.

Although the apparent duration of the peak in the chromatogram is less than 30 seconds, structural determination procedures are simple and sodiated ions are abundant, such that the entire CID spectrum measurement with a favourable signal-to-noise ratio can be performed three times within 30 seconds. The detection limit of our method for trisaccharide was estimated to be lower than 0.33 nmole from the amount of panose used in Fig. 8 (10 μl injection of 10^−4^ M solution for three times of MS^n^ spectrum measurements). The successful structural determination in this study indicates the high capability of this method for the *in situ* structural determination of oligosaccharides through chromatograms.

### Comparison to other MS methods

Currently, the determination of linkages, anomeric configurations, and the branching location of oligosaccharides represents a major limitation in carbohydrate research. Several mass spectrum approaches have been developed to determine the structures of carbohydrates. A commonly used method is the mass spectra of permethylated carbohydrates^13^. This method requires the permethylation of carbohydrates prior to mass spectrum measurement. A fraction of sample may be lost due to the incomplete permethylation and during the extraction of the permethylated carbohydrates. This method only provides the information of linkages.

Another method is the current de novo structural determination for oligosaccharides developed by Bendiak *et al*.^34–36^. It provides the information of monosaccharide constitute, linkages, and anomeric configuration. However, the demonstrating experiment shows that it takes 11 hours to obtained good signal-to-noise ratio mass spectra from a tetrasaccharide^36^. These spectra only provide the information of two monosaccharide constitutes at the nonreducing side, and the linkages and anomeric configurations of two glycosidic bonds. One glycosidic bond and two monosaccharide constitutes at the reducing side cannot be determined. In addition, Bendiak’s method only works for linear oligosaccharides.

The third method is to build glycan mass spectrum libraries. Mass spectra of unknown sample are compared to the spectra in glycan library for structural identification. However, building a complete glycan MS library is time consuming, considering that the high number of carbohydrate isomers (e.g., 10^12^ isomers for an oligosaccharide containing six hexoses) of a given chemical formula. Most of these isomers are not available at this moment and the synthesis of each isomer takes weeks for an experienced chemist. Even if all isomers are available, they are not likely to be distinguishable from each other by a single mass spectrum. Mass spectra obtained from multiple-stage tandem mass spectrometry are necessary in the structural determination. If each stage generates 10 fragments, there are more than 100 spectra in a MS^4^ experiment. The measurement of 100 spectra for a given isomer is impractical, not to mention that most of the spectra are similar or identical which are not useful in the structural identification. Hence, a guild line to choose the critical fragments for MS^n^ measurement is needed.

In this study, we demonstrated a simple and rapid method with high sensitivity for the structural determination of underivatised glucose oligosaccharides. Our method can determine the linkages, branch location, and anomeric configurations for both linear and branched oligosaccharides. Currently, other methods can only determine the molecular weights and part of the structures during the short appearance period of each oligosaccharide in liquid chromatography. In our method, the CID spectra with good S/N ratio can be obtained within a very short period of time, and the number of CID spectra requires for the structure determination is minimized by the logical procedure we developed. These advantages enable us to *in situ* determine the structure of each oligosaccharide separated from liquid chromatography.

Our method provides a simple logical procedure to determine the structural decisive fragments for MS^n^ measurement and only the disaccharide database is required. It greatly reduces the effort in building the glycan mass spectrum library for structural identification. The concept of this method can be extended to larger glucose oligosaccharides. Because the structural similarity of galactose, mannose, and glucose, the same concept can be applied to galactose- and mannose- oligosaccharides if the corresponding disaccharide database is available. The applications to mannose are demonstrated in a separate report^46^.

The drawback of our method is that it does not work for mixture of glycans if the glycans happen to have the same molecular weight. This is also the limitation of current structural determination methods using MS. The probability of the coincidence that glycans with the same molecular weight happen to be in a single CE or HPLC eluent peak is small. Consequently, combination of CE or HPLC with mass spectrometer in our method can conquer most of the difficulty in the analysis of mixture, although such combination may require larger amount of sample.

## Methods

### HPLC-ESI-MS^n^

The CID spectra of disaccarhides in the database were measured by using a heated electrospray ionization (HESI-II) probe with an Ion Max housing and a linear ion trap mass spectrometer (LTQ XL, Thermo Fisher Scientific, Waltham, MA USA) coupled with an HPLC system (Dionex Ultimate 3000, Thermo Fisher Scientific, Waltham, MA USA) in the positive mode. The entire HPLC and mass spectrometer system is controlled by using Dinoex Chromatography MS Link 2.14, Chromeleon Version 6.80 SR13, LTQ Tune Plus Version 2.7.0.1103 SP1, and Thermo Xcalibur 2.2 SP1.48 software from Thermo Fisher Scientific. No customization of these instruments was made.

Liquid chromatography separation of all disaccharides was achieved using a Hypercarb (100 × 2.1 mm^2^, Thermo Fisher Scientific, Waltham, MA USA) column with a particle size of 3 µm operated in the multistep gradient mode at 25 °C. The mobile phase comprised (A) 0.1% (v/v%) aqueous formic acid containing 1 × 10^−4^ M NaCl and (B) HPLC-grade acetonitrile. The multistep gradient mode conditions were as follows: *t* = 0 min, A: 100%, B: 0%; *t* = 1 min, A: 100%, B: 0%; *t* = 21 min, A: 90%, B: 10%; *t* = 21.1 min, A: 100%, B: 0%. For laminaribiose, the mobile phase gradient was as follows: *t* = 0 min, A: 95%, B: 5%; *t* = 1 min, A: 95%, B: 5%; *t* = 26 min, A: 94%, B: 6%. Samples were prepared in ultrapure water at a concentration of 1 × 10^−4^ M. The injection volume of the sample was 10 µL, and the mobile phase flow rate was 300 µL/min. The column eluate was directly infused into the ESI source without any postcolumn addition. The MS conditions were optimised using the built-in semiautomatic tuning procedure in the Xcalibur software. The ESI source was operated at a temperature of 280 °C with 30 units of sheath gas flow and 10 units of auxiliary gas flow. The ion spray voltage was 4.00 kV, and the transfer capillary temperature was 280 °C. The capillary voltage was 80 V, and the tube lens voltage was 150 V. Helium gas was used as a buffer gas for the ion trap as well as a collision gas in CID. The pressure of He gas at the output of regulator connected to gas cylinder was set at the specification (40 psi). The pressure measured by the ion gauge in the vacuum chamber of mass spectrometer was 0.9 × 10^−5^ Torr. The MS^n^ experiments were performed at an activation Q value of 0.25, an activation time of 30 ms, normalised collision energy 25% for standard spectra of disaccharide database, and normalised collision energy 20–100% with 10% increment for test spectra. The numbers of standard and test spectra taken for database and the calculations of uncertainty were described in Supplementary Information. The number of ions was regulated by injection time (5 ms) or automatic gain control (1 × 10^5^ for full scan, and 1 × 10^4^ for MS^n^). The precursor ion isolation width was set to 1 or 2 u. No difference in spectra was observed for the change of isolation width. MS^n^ of panose in Fig. 8 were measured using the same conditions of disaccharides. Spectra were analysed by in-house code. Threshold of spectra was set to be 0.01.

### ESI-MS^n^

All oligosaccharide MS^n^ (except Fig. 8) were obtained using the same mass spectrometer under the same operation conditions as for disaccharides, except that HPLC was not used, the ESI source was operated at a temperature of 35 °C, and CID was performed only at normalised collision energy of 30%. Samples were prepared in 50% (v/v%) HPLC-grade methanol and ultrapure water at a concentration of 1 × 10^−4^ M. Sodium chloride was added to the sample solution at a concentration of 1 × 10^−4^ M. A total of 1 or 2 minutes spectral acquisition time were accumulated for each MS spectrum in Figs 4–6. They are the average of 250 or 500 microscans.

### Data Availability

All data generated or analysed during this study are included in this published article (and its Supplementary Information and Supplementary Data files).

## Electronic supplementary material

Supplementary information

Supplementary Dataset 1
