# Supplementary material for: Simple Method for De Novo Structural Determination of Underivatised Glucose Oligosaccharides
Source: Sci Rep. 2018 Apr 3;8:5562. doi: 10.1038/s41598-018-23903-4 (PMC5882935; doi:10.1038/s41598-018-23903-4)
Supplement: Supplementary file 1 — Supplementary information [file 41598_2018_23903_MOESM1_ESM.docx]

**Supplementary Information**

**Simple Method for *De Novo* Structural Determination of Underivatized Glucose Oligosaccharides**

Hsu Chen Hsu^1^, Chia Yen Liew^1^, Shih-Pei Huang^1,2^, Shang-Ting Tsai^1^, and Chi-Kung Ni*^1,3^

1. Materials …………………………………………………………. S2

2. Similarity calculations........…….………………………………… S2

2.1 Database ……………………………………………………… S3

2.2 Oligosaccharides ……………………………………………... S6

(a) Panose…………………………………………………….. S6

(b) β-Glc-(1→3)-β-Glc-(1→4)-Glc……………………......... S7

(e) Isopanose…………………………………………………. S9

(f) Cellotetraose……………………………………………… S9

3. Predicted fragmentation pattern of trisaccharides………………… S9

4. Built in logical procedure………………………………………..... S11

5. In-house codes for intensity and similarity calculations……..…… S11

6. References ………………………………………………………. . S19

**1. Materials**

Kojibiose (99.1 %), laminaribiose (95.0 %), isopanose, maltotriose monohydrate (98 %), panose (>98 %), and cellotetraose (95 %) were obtained from Carbosynth, Ltd. (Campton Berkshire, UK); maltose monohydrate (>98 %) and isomaltose (>97.0 %) were purchased from Tokyo Chemical Industry Co., Ltd. (Tokyo, Japan); nigerose (≧94 %), cellobiose (≧98 %), cellotriose (≧93 %), and isomaltotriose (≧98 %) were purchased from Sigma Aldrich, Inc. (St. Louis, MO, USA); α-sophorose was obtained from ChromaDex, Inc. (Irvine, CA, USA), and β-gentiobiose (98 %-99.8 %) were purchased from Nacalai Tesque, Inc. (Kyoto, Japan). All saccharides were used without further purification. HPLC-grade methanol was purchased from J. T. Baker, Avantor Performance Materials, LLC. (Center Valley, PA, USA). The ultrapure water was obtained using the Simplicity water purification system (Merck Millipore, Billerica, MA, USA). Sodium chloride was purchased from Arcos Organics (Geel, Belgium).

**2. Similarity calculations**

The structures of the disaccharides produced from oligosaccharides were assigned by comparing the CID spectra to the spectra in the database. The assignments were validated by calculating the similarity for spectral matching. Two methods for calculating the spectral similarity were used^1,2^.

$$\mathrm{Method}\left( I \right): Similarity= \frac{\sum\sqrt{{I_{m}^{s}I}_{m}^{t}}}{\sqrt{\sum I_{m}^{s}\sum I_{m}^{t}}}$$

$$\mathrm{Method}\left( \mathrm{II} \right): Similarity= \frac{\sum{I_{m}^{s}I}_{m}^{t}}{\sqrt{\sum{(I}_{m}^{s})^{2}\sum{(I}_{m}^{t})^{2}}}$$

where I^s^_m_ and I^t^_m_ are the normalized intensities of ion m for the standard spectra and test spectra, respectively. Spectral similarity has values between 0 and 1. If two spectra are identical, the spectral similarity is 1. In general, a large similarity suggests close similarity between the two spectra, indicating high structural similarity. We found that method (I) works best when the number of peaks in each spectrum are more than two, and method (II) works best for only two peaks in each spectrum.

We first applied the calculations to the disaccharide spectra in the database. We demonstrated that the spectra obtained from the disaccharides of the same structures have a high similarity, and those from the disaccahrides of different structures have a low similarity. Subsequently, we applied these methods to oligosaccharides for structural assignment.

**2.1 Database**

The spectra of the database are the standard spectra in Table S1-3. To find out the reliability of these spectra, a set of test spectra was used to calculate the spectral similarity of standard spectra and test spectra. The standard CID spectra of disaccharides are the average of 10 repeated experiments (only 7 for laminaribiose) at 25% of the normalized collision energy. Three to eight spectra were taken in each experiment, depending on the duration time of each peak shown in chromatography. Each spectrum represents an average of 5 microscans. The test spectra include every single spectrum obtained at the normalized collision energy ranging from 20% to 100% with 10% increment. Two to 5 experiments for each collision energy were measured. Similar to the standard spectra, three to eight spectra were taken in each experiment, depending on the duration time of each peak shown in chromatography. Each test spectrum is an average of 5 microscans. These standard and test spectra were obtained by three persons in 6 months.

Because the test spectra were not the average of several spectra, they have relatively large fluctuation in relative ion intensities. They are used to imitate the spectra obtained in practical applications. In the calculations of similarity, the disaccharide spectrum matching process is divided into three steps for identifying the linkage types, anomeric configuration of glycosidic bond, and anomeric configuration at the reducing end, respectively. In the first step, method (I) and ions m/z 305, 275, 245 were used for linkage determination. The same types of linkages have a high similarity (0.99–1), as shown in the diagonal elements in Table S1. Different types of linkages, as shown in the off-diagonal elements in Table S1, have a low similarity. The results suggest that the linkage types can be distinguished using the calculated similarity and that the fingerprints of spectra are not sensitive to the collision energy. A range of numbers in Tables indicates the maximum and minimum values of similarity calculated from the test spectra and standard spectra. They represent the uncertainly of the similarity. If it is one number, e.g., 1.00, it means the uncertainty is smaller than 0.01.

Table S1. Similarity of linkage calculated by using method (I).

|  |  | Test Spectra | | | |
| --- | --- | --- | --- | --- | --- |
|  |  | 1-2 | 1-3 | 1-4 | 1-6 |
| Standard | 1-2 | 1.00 | 0.07-0.26 | 0.12-0.26 | 0.22-0.37 |
|  | 1-3 | 0.07-0.25 | 0.99-1.00 | 0.19-0.31 | 0.54-0.62 |
|  | 1-4 | 0.12-0.26 | 0.18-0.36 | 0.99-1.00 | 0.89-0.95 |
|  | 1-6 | 0.25-0.36 | 0.55-0.67 | 0.90-0.95 | 1.00 |

The anomeric configuration of glycosidic bond was determined in the second step by using method (II). Ions include m/z 203 and 245 for 1→2 linkage, 203 and 275 for 1→3 linkage, 203 and 305 for 1→4 linkage, and 203 and 245 for 1→6 linkage. The results are listed in Table S2.

Table S2. Similarity of glycosidic bond anomeric configuration calculated by using method (II).

|  |  | Test spectra | | | | | | | |
| --- | --- | --- | --- | --- | --- | --- | --- | --- | --- |
|  |  | α1-2 | β1-2 | α1-3 | β1-3 | α1-4 | β1-4 | α1-6 | β1-6 |
| Standard | α1-2 | 0.97-  1.00 | 0.71-  0.81 |  |  |  |  |  |  |
|  | β1-2 | 0.63-  0.79 | 1.00 |  |  |  |  |  |  |
|  | α1-3 |  |  | 0.98-  1.00 | 0.53-  0.78 |  |  |  |  |
|  | β1-3 |  |  | 0.52-  0.74 | 0.99-  1.00 |  |  |  |  |
|  | α1-4 |  |  |  |  | 0.94-  1.00 | 0.65-  0.87 |  |  |
|  | β1-4 |  |  |  |  | 0.65-  0.89 | 1.00 |  |  |
|  | α1-6 |  |  |  |  |  |  | 0.96-  1.00 | 0.64  -0.81 |
|  | β1-6 |  |  |  |  |  |  | 0.60-  0.80 | 0.99  -1.00 |

Method (II) was also used in the third step to determine the anomeric configuration at the reducing end. Ions m/z 275 and 347 for 1→3 linkage, ions m/z 305 and 347 for 1→4 linkage, and ion m/z 245 and 347 for 1→6 linkage were used for similarity calculations. The results are listed in Table S3. The large values of similarity for the same anomeric configuration and small values for different anomeric configurations provide highly confident assignments for the anomeric configuration.

Table S3. Similarity of anomeric configuration at reducing end calculated by using method (II).

|  |  | Test spectra | | | |
| --- | --- | --- | --- | --- | --- |
|  |  | α1-3α | α1-3β | β1-3α | β1-3β |
| Standard | α1-3α | 1.00 | 0.49-0.67 |  |  |
|  | α1-3β | 0.46-0.55 | 0.97-1.00 |  |  |
|  | β1-3α |  |  | 1.00 | 0.25-0.37 |
|  | β1-3β |  |  | 0.29-0.36 | 0.99-1.00 |
|  |  | α1-4α | α1-4β | β1-4α | β1-4β |
|  | α1-4α | 1.00 | 0.85-0.90 |  |  |
|  | α1-4β | 0.83-0.90 | 1.00 |  |  |
|  | β1-4α |  |  | 1.00 | 0.84-0.89 |
|  | β1-4β |  |  | 0.85-0.89 | 1.00 |
|  |  | α1-6α | α1-6β | β1-6α | β1-6β |
|  | α1-6α | 0.98-1.00 | 0.81-0.96 |  |  |
|  | α1-6β | 0.83-0.97 | 0.98-1.00 |  |  |
|  | β1-6α |  |  | 0.99-1.00 | 0.80-0.89 |
|  | β1-6β |  |  | 0.80-0.90 | 0.99-1.00 |

**2.2 Oligosaccharides**

**(a) Calculated similarities of CID spectra of Panose**

Table S4(a). Similarity of Figure 4(g).

|  | 1-2 | 1-3 | 1-4 | | 1-6 |
| --- | --- | --- | --- | --- | --- |
| linkage | 0.16 | 0.24 | 1.00 | | 0.91 |
| Anomeric configuration of glycosidic bond |  |  | α | 1.00 |  |
|  |  |  | β | 0.82 |  |

Table S4(b). Similarity of Figure 4(h).

|  | 1-2 | 1-3 | 1-4 | 1-6 | |
| --- | --- | --- | --- | --- | --- |
| linkage | 0.27 | 0.63 | 0.93 | 1.00 | |
| Anomeric configuration of glycosidic bond |  |  |  | α | 1.00 |
|  |  |  |  | β | 0.53 |
| Anomeric configuration at reducing end |  |  |  | α | 1.00 |
|  |  |  |  | β | 0.94 |

Table S4(c). Similarity of Figure 4(e).

|  | 1-2 | 1-3 | 1-4 | | 1-6 |
| --- | --- | --- | --- | --- | --- |
| linkage | 0.15 | 0.23 | 1.00 | | 0.91 |
| Anomeric configuration of glycosidic bond |  |  | α | 1.00 |  |
|  |  |  | β | 0.83 |  |

Table S4(d). Similarity of Figure 4(f).

|  | 1-2 | 1-3 | 1-4 | 1-6 | |
| --- | --- | --- | --- | --- | --- |
| linkage | 0.28 | 0.63 | 0.93 | 1.00 | |
| Anomeric configuration of glycosidic bond |  |  |  | α | 1.00 |
|  |  |  |  | β | 0.70 |
| Anomeric configuration of reducing end |  |  |  | α | 1.00 |
|  |  |  |  | β | 0.93 |

**(b) Calculated similarities of CID spectra of β-Glc-(1→3)-β-Glc-(1→4)-Glc**

Table S5(a). Similarity of Figure 5(h).

|  | 1-2 | 1-3 | | 1-4 | 1-6 |
| --- | --- | --- | --- | --- | --- |
| linkage | 0.16 | 1.00 | | 0.30 | 0.633 |
| Anomeric configuration of glycosidic bond |  | α | 0.66 |  | |
|  |  | β | 1.00 |  |  |
| Anomeric configuration of reducing end |  | α | 0.15 |  |  |
|  |  | β | 0.99 |  |  |

Table S5(b). Similarity of Figure 5(f).

|  | 1-2 | 1-3 | | 1-4 | 1-6 |
| --- | --- | --- | --- | --- | --- |
| linkage | 0.19 | 1.00 | | 0.33 | 0.65 |
| Anomeric configuration of glycosidic bond |  | α | 0.66 |  | |
|  |  | β | 1.00 |  |  |
| Anomeric configuration of reducing end |  | α | 0.18 |  |  |
|  |  | β | 0.99 |  |  |

Table S5(c). Similarity of Figure 5(g).

|  | 1-2 | 1-3 | 1-4 | | 1-6 |
| --- | --- | --- | --- | --- | --- |
| linkage | 0.22 | 0.25 | 1.00 | | 0.92 |
| Anomeric configuration of glycosidic bond |  |  | α | 0.86 |  |
|  |  |  | β | 1.00 |  |

Table S5(d). Similarity of Figure 5(e).

|  | 1-2 | 1-3 | 1-4 | | 1-6 |
| --- | --- | --- | --- | --- | --- |
| linkage | 0.21 | 0.25 | 1.00 | | 0.91 |
| Anomeric configuration of glycosidic bond |  |  | α | 0.86 |  |
|  |  |  | β | 1.00 |  |

**(c) Calculated similarities of CID spectra of Isopanose**

Table S6. Similarity of Figure 6(d).

|  | 1-2 | 1-3 | 1-4 | 1-6 | |
| --- | --- | --- | --- | --- | --- |
| linkage | 0.34 | 0.67 | 0.90 | 1.00 | |
| Anomeric configuration of glycosidic bond |  |  |  | α | 1.00 |
|  |  |  |  | β | 0.82 |

**(d) Calculated similarities of CID spectra of Cellotetraose**

Table S7(a). Similarity of Figure 6(h).

|  | 1-2 | 1-3 | 1-4 | | 1-6 |
| --- | --- | --- | --- | --- | --- |
| linkage | 0.19 | 0.33 | 1.00 | | 0.94 |
| Anomeric configuration of glycosidic bond |  |  | α | 0.86 |  |
|  |  |  | β | 1.00 |  |
| Anomeric configuration at reducing end |  |  | α | 0.82 |  |
|  |  |  | β | 1.00 |  |

Table S7(b). Similarity of Figure 6(i).

|  | 1-2 | 1-3 | 1-4 | | 1-6 |
| --- | --- | --- | --- | --- | --- |
| linkage | 0.23 | 0.31 | 1.00 | | 0.94 |
| Anomeric configuration of glycosidic bond |  |  | α | 0.86 |  |
|  |  |  | β | 1.00 |  |
| Anomeric configuration at reducing end |  |  | α | 0.85 |  |
|  |  |  | β | 1.00 |  |

**3. Fragmentation patterns of sodiated trisaccharides**

Table S8. Low-energy CID fragments of sodiated trihexoses (m/z = 527). Fragments produced in MS^2^ and MS^3^ are in red and green, respectively.

|  | 185 | **V** | **V** | **V** | **V** | **V** | **V** | **V** | **V** | **V** | **V** | **V** | **V** | 185 | **V** | **V** | **V** | **V** | **V** | **V** |
| --- | --- | --- | --- | --- | --- | --- | --- | --- | --- | --- | --- | --- | --- | --- | --- | --- | --- | --- | --- | --- |
|  | 203 | **V** | **V** | **V** | **V** | **V** | **V** | **V** | **V** | **V** | **V** | **V** | **V** | 203 | **V** | **V** | **V** | **V** | **V** | **V** |
|  | 245 |  |  |  |  |  |  |  |  |  |  |  |  | 245 |  |  | **V** |  | **V** |  |
|  | 275 |  |  |  |  |  |  |  |  |  |  |  |  | 275 |  | **V** | **V** |  |  |  |
|  | 305 |  |  |  |  |  |  |  |  |  |  |  |  | 305 |  |  | **V** |  | **V** |  |
|  | 347 | **V** | **V** | **V** | **V** | **V** | **V** | **V** | **V** | **V** | **V** | **V** | **V** | 347 | **V** | **V** | **V** | **V** | **V** | **V** |
| **365→** | 245 | **V** | **V** | **V** | **V** | **V** | **V** | **V** | **V** | **V** |  |  | **V** | 245 | **V** | **V** | **V** |  | **V** | **V** |
|  | 275 | **V** | **V** | **V** |  | **V** | **V** | **V** |  | **V** |  | **V** |  | 275 | **V** | **V** | **V** | **V** |  | **V** |
|  | 305 | **V** | **V** | **V** | **V** | **V** |  | **V** |  | **V** | **V** |  |  | 305 | **V** | **V** | **V** | **V** | **V** |  |
|  | 347 | **V** | **V** | **V** | **V** | **V** | **V** | **V** | **V** | **V** | **V** | **V** |  | 347 | **V** | **V** | **V** | **V** | **V** | **V** |
|  | 365 | **V** | **V** | **V** | **V** | **V** | **V** | **V** | **V** | **V** | **V** | **V** | **V** | 365 | **V** | **V** | **V** | **V** | **V** | **V** |
| **407→** | 245 | **V** | **V** |  |  |  |  | **V** | **V** |  |  |  |  | 245 |  |  |  |  |  |  |
|  | 347 | **V** |  |  |  |  |  | **V** |  |  |  |  |  | 347 |  |  |  |  |  |  |
|  | 365 | **V** | **V** |  |  |  |  | **V** | **V** |  |  |  |  | 365 |  |  |  |  |  |  |
|  | 407 | **V** | **V** |  |  |  |  | **V** | **V** |  |  |  |  | 407 |  |  |  |  |  |  |
| **437→** | 275 | **V** | **V** |  |  | **V** | **V** |  |  |  |  |  |  | 275 |  |  |  |  |  | **V** |
|  | 347 | **V** |  |  |  | **V** |  |  |  |  |  |  |  | 347 |  |  |  |  |  |  |
|  | 365 | **V** | **V** |  |  | **V** | **V** |  |  |  |  |  |  | 365 |  |  |  |  |  |  |
|  | 437 | **V** | **V** |  |  | **V** | **V** |  |  |  |  |  |  | 437 |  |  |  |  |  | **V** |
| **467→** | 245 |  |  |  |  |  |  |  |  |  |  |  |  | 245 | **V** |  |  |  |  |  |
|  | 305 | **V** | **V** | **V** | **V** |  |  |  |  |  |  |  |  | 305 | **V** |  |  |  |  |  |
|  | 347 | **V** |  | **V** |  |  |  |  |  |  |  |  |  | 347 |  |  |  |  |  |  |
|  | 365 | **V** | **V** | **V** | **V** |  |  |  |  |  |  |  |  | 365 |  |  |  |  |  |  |
|  | 407 | **V** | **V** | **V** | **V** |  |  |  |  |  |  |  |  | 407 |  |  |  |  |  |  |
|  | 467 | **V** | **V** | **V** | **V** |  |  |  |  |  |  |  |  | 467 | **V** |  |  |  |  |  |
| **509→** | 347 | **V** | **V** | **V** | **V** | **V** | **V** |  |  |  |  |  |  | 347 | **V** | **V** |  | **V** |  |  |
|  | 365 | **V** | **V** | **V** | **V** | **V** | **V** |  |  |  |  |  |  | 365 |  |  |  |  |  |  |
|  | 509 | **V** | **V** | **V** | **V** | **V** | **V** |  |  |  |  |  |  | 509 | **V** | **V** |  | **V** |  |  |
|  | linear | 1-x, 1-6 | 1-2, 1-6 | 1-x, 1-4 | 1-2, 1-4 | 1-x, 1-3 | 1-2,1-3 | 1-x, 1-2 | 1-2, 1-2 | 1-6, 1-1 | 1-4, 1-1 | 1-3, 1-1 | 1-2, 1-1 | branched | 1-6, 1-4 | 1-6, 1-3 | 1-6, 1-2 | 1-4, 1-3 | 1-4, 1-2 | 1-3, 1-2 |

**4. Built in logical procedure**

We wrote the following procedures in Thermo Xcalibur 2.2 SP1.48 software (Thermo Fisher Scientific, Waltham, MA USA) to obtain the spectra in Figure 8. Not all spectra are illustrated in Figure 8.

1. Full scan
2. 527→
3. 527→509→365→
4. 527→467→365→
5. 527→439→365→
6. 527→365→
7. 527→509→
8. 527→467→
9. 527→437→

**5. In-house codes for intensity and similarity calculations**

There are two in-house codes. One calculates the areas of selected m/z peaks from raw data and generates the intensities of the selected peaks. The second code calculates the similarity for spectrum matching. Both codes are written using Matlab.

5.1 Intensity

close all;

clear all;

tic

%----------------------------------------------------------------------

%-------------Select import data---------------------------------------

%----------------------------------------------------------------------

% Import raw data from Origin files

[Fn, Pn] = uigetfile('*.opj','Select a dat file');

fullFileName = fullfile(Pn, Fn);

%----------------------------------------------------------------------

%-------------Loading MS raw data--------------------------------------

%----------------------------------------------------------------------

A=importOrigin([fullFileName]);

NumSpectra=size(A,2);

for i=1:NumSpectra;

Fullfile=A(i);

File1=Fullfile.name;

File2=split(File1,'Name(');

FileName{i}=(strrep(File2(2,1),')',''));

FileNum=split(FileName{i},'',2);

FileNumber{i}=str2double(strcat(FileNum(5),FileNum(6),FileNum(7)));

Spectrum{i}=Fullfile.xy;

end

%----------------------------------------------------------------------

%-------------Calculating the Intensity ratio of all peaks-------------

%----------------------------------------------------------------------

% Integrate intensity with peakwidth=1, cutting thrshold @ 0.01

peakWidth=1;

IntPeaks=0.01;

% This will connect to an existing instance of Origin, or create a new one if none exist

originObj=actxserver('Origin.ApplicationSI');

% Make the Origin session visible

invoke(originObj, 'Execute', 'doc -mc 1;');

% Clear "dirty" flag in Origin to suppress prompt for saving current project

invoke(originObj, 'IsModified', 'false');

% Select m/z for peak integration

for i=1:NumSpectra;

Integrate=[];

if FileNumber{i}==365

peakpeaks=[185, 203, 245, 275, 305, 335, 347, 365];

elseif FileNumber{i}==367;

peakpeaks=[185, 203, 205, 245, 247, 275, 277, 305, 307, 335, 337, 347, 349, 367];

elseif FileNumber{i}==347;

peakpeaks=[167, 185, 201, 203, 245, 257, 257, 269, 271, 275, 287, 289, 299, 317, 329];

elseif FileNumber{i}==305;

peakpeaks=[143, 185, 203, 245, 275, 287];

elseif FileNumber{i}==275;

peakpeaks=[113, 185, 203, 257];

elseif FileNumber{i}==187;

peakpeaks=[67, 69, 79, 81, 91, 93, 97, 99, 109, 111, 113, 115, 117, 121, 123, 127, 129, 139, 141, 145, 147, 151, 153, 157, 159, 169, 171, 187, 189];

elseif FileNumber{i}==169;

peakpeaks=[67, 69, 79, 81, 91, 93, 97, 99, 109, 111, 113, 115, 117, 121, 123, 127, 129, 139, 141, 145, 147, 151, 153, 157, 159, 169];

elseif FileNumber{i}==127;

peakpeaks=[67, 69, 79, 81, 91, 93, 97, 99, 109, 111, 113, 115, 117, 121, 123, 127];

end

peaksRange = [peakpeaks'-peakWidth, peakpeaks'+peakWidth];

for j=1:size(peaksRange,1);

[vMin,iMin] = min(abs(Spectrum{i}(:,1)-peaksRange(j,1)));

[vMax,iMax] = min(abs(Spectrum{i}(:,1)-peaksRange(j,2)));

[vMaxI,Numa] = max(Spectrum{i}(iMin:iMax,2));

if vMaxI<IntPeaks;

locsPeaks=Spectrum{i}(iMin:iMax,2);

else

[~, pMin] = min(abs(Spectrum{i}(:,1)-(Spectrum{i}(iMin+Numa-1,1)-peakWidth)));

[~, pMax] = min(abs(Spectrum{i}(:,1)-(Spectrum{i}(iMin+Numa-1,1)+peakWidth)));

locsPeaks=Spectrum{i}(pMin:pMax,2);

end

Integrate(j,:)=sum(locsPeaks,1);

end

IntRat=100*Integrate/max(Integrate);

IntRatio=horzcat(peakpeaks',IntRat);

newDataBookName=char(FileName{i});

invoke(originObj, 'CreatePage', '2',newDataBookName,'origin');

% Name the X axis as m/z and Y axis as Intensity

invoke(originObj, 'Execute', strcat('col(A)[L]$ = m/z; col(B)[L]$ = ',newDataBookName,''));

invoke(originObj, 'Execute', 'col(B)[U]$ = %');

% import data into the open Worksheet of OriginLab

invoke(originObj, 'PutWorksheet', newDataBookName, IntRatio);

end

%----------------------------------------------------------------------

%-------------Is substraction necessary ?------------------------------

%----------------------------------------------------------------------

prompt = {'Is Substraction of Standard necessary ?'};

name='Loading Standard';

numlines=1;

defaultanswer={'N'};

options.Resize='on';

options.WindowStyle='normal';

answer=inputdlg(prompt,name,numlines, defaultanswer,options);

Nextstep = answer{1};

yes=strcat(Nextstep);

if yes ==char('N');

clear originObj;

return;

elseif yes == char('n');

clear originObj;

return;

elseif isempty(yes);

clear originObj;

return;

else

end

%----------------------------------------------------------------------

%-------------Select Standard database---------------------------------

%----------------------------------------------------------------------

[Fn1, Pn1] = uigetfile('*.dat','Select a dat file');

[pathstr,Filestr,ext] = fileparts(Fn1);

fullFileName2 = fullfile(Pn1, Fn1);

Stand=importdata([fullFileName2]);

invoke(originObj, 'CreatePage', '2','Standard','origin');

% import data into the open Worksheet of OriginLab

invoke(originObj, 'PutWorksheet', 'Standard', Stand.data);

invoke(originObj, 'Save', fullFileName);

clear originObj;

tic;

5.2 Similarity

close all;

clear all;

tic

%----------------------------------------------------------------------%-------------Select import data--------------------------------------

%----------------------------------------------------------------------% Import integrated data from Origin files

[Fn, Pn] = uigetfile('*.opj','Select a dat file');

fullFileName = fullfile(Pn, Fn);

% This will connect to an existing instance of Origin, or create a new one if none exist

originObj=actxserver('Origin.ApplicationSI');

% Make the Origin session visible

invoke(originObj, 'Execute', 'doc -mc 1;');

% Clear "dirty" flag in Origin to suppress prompt for saving current project

invoke(originObj, 'IsModified', 'false');

A=importOrigin([fullFileName]);

NumSpectra=size(A,2);

% Select peak for similarity calculation

for i=1:NumSpectra;

Fullfile=A(i);

File1=Fullfile.name;

File2=split(File1,'Name(');

FileName =(strrep(File2(2,1),')',''));

FileNum=split(FileName ,'',2);

FileNumber=str2double(strcat(FileNum(5),FileNum(6),FileNum(7)));

IntRatio =Fullfile.xy;

clear Stand Simil CSimil Similarity T;

if FileNumber == 365;

T{1}=[245, 275, 305];

T{2}=[203, 245, 347];

T{3}=[203, 275, 347];

T{4}=[203, 305, 347];

T{5}=[203, 245];

T{6}=[203, 275];

T{7}=[203, 305];

T{8}=[245, 347];

T{9}=[275, 347];

T{10}=[305, 347];

elseif FileNumber == 367;

T{1}=[245, 275, 305];

T{2}=[203, 245, 347];

T{3}=[203, 275, 347];

T{4}=[203, 305, 347];

T{5}=[203, 245];

T{6}=[203, 275];

T{7}=[203, 305];

T{8}=[245, 347];

T{9}=[275, 347];

T{10}=[305, 347];

elseif FileNumber == 347;

T{1}=[167, 201, 185, 203, 245, 257, 257, 269, 271, 275, 287, 289, 299, 317, 329];

T{2}=[167, 201, 203, 245, 257, 257, 269, 271, 275, 287, 289, 299, 317, 329];

T{3}=[167, 201, 203, 245, 257, 257, 269, 271, 275, 287, 289, 299, 317, 329];

elseif FileNumber == 305;

T{1}=[275, 287];

T{2}=[143, 287];

T{3}=[143, 275];

elseif FileNumber == 275;

T{1}=[203, 257];

T{2}=[185, 257];

T{3}=[113, 203];

elseif FileNumber == 187;

T{1}=[127, 169];

elseif FileNumber == 169;

T{1}=[67, 91, 97];

T{2}=[81, 91, 93];

T{3}=[81, 91, 97];

elseif FileNumber == 127;

T{1}=[67, 91];

else

return

end

%----------------------------------------------------------------------

%-------------Loading Standard database--------------------------------

%----------------------------------------------------------------------

StandFile=strcat('D:\Lab320\Thermo\Disaccharides\Man-Man\365 database with HPLC separation\Real sampe\Mannobiose_database_',num2str(FileNumber),'_NCE30.dat');

Stand=importdata([StandFile]);

%----------------------------------------------------------------------

%-------------Calculating Similarity-----------------------------------

%----------------------------------------------------------------------

for j=1:size(T,2);

Selepeaks=T{j};

Peakindex=(ismember(IntRatio(:,1)',Selepeaks))';

Peakindexstand=(ismember(Stand.data(:,1),Selepeaks))';

CSimiA=IntRatio(:,2)'.*Peakindex';

SimiA=CSimiA.^0.5;

Numstandard=size(Stand.data,2)-1;

for i=1:Numstandard;

CSimiS=(Stand.data(:,i+1).*Peakindexstand')';

SimiS=CSimiS.^0.5;

Simil(j,i)=1-pdist([SimiA;SimiS],'cosine');

CSimil(j,i)=1-pdist([CSimiA;CSimiS],'cosine');

end

Peaks(j,2:size(T{j},2)+1)=T{j};

end

Similar=horzcat(Peaks,Simil);

SumSimilar=sum(Similar,1);

CSimilar=horzcat(Peaks,CSimil);

SumCSimilar=sum(CSimilar,1);

Similarity=vertcat(Similar,SumSimilar,CSimilar,SumCSimilar);

Similarity=roundn(Similarity,-2);

newDataBookName=char(strcat('S',FileName));

% Import to Origin

invoke(originObj, 'CreatePage', '2', newDataBookName,'origin');

% Rename the active worksheet

invoke(originObj, 'Execute', 'wks.name$ = Similarity');

% Name the X axis as m/z and Y axis as Intensity

invoke(originObj, 'Execute', strcat('col(A)[L]$ = method; col(B)[L]$ = picking peaks; col(C)[L]$ = picking peaks; col(D)[L]$ = picking peaks; col(E)[L]$ = score'));

invoke(originObj, 'Execute', strcat('col(E)[C]$ = Man-a12-Man-1; col(F)[C]$ = Man-a12-Man-2; col(G)[C]$ = Man-b12-Man-1; col(H)[C]$ = Man-b12-Man-2;'));

invoke(originObj, 'Execute', strcat('col(I)[C]$ = Man-a13-Man-a; col(J)[C]$ = Man-a13-Man-b; col(K)[C]$ = Man-b13-Man-a; col(L)[C]$ = Man-b13-Man-b;'));

invoke(originObj, 'Execute', strcat('col(M)[C]$ = Man-a14-Man-a; col(N)[C]$ = Man-a14-Man-b; col(O)[C]$ = Man-b14-Man-a; col(P)[C]$ = Man-b14-Man-b;'));

invoke(originObj, 'Execute', strcat('col(Q)[C]$ = Man-a16-Man-1; col(R)[C]$ = Man-a16-Man-2; col(S)[C]$ = Man-b16-Man-1; col(T)[C]$ = Man-b16-Man-2;'));

% import data into the open Worksheet of OriginLab

invoke(originObj, 'PutWorksheet', newDataBookName, Similarity);

end

invoke(originObj, 'Save', fullFileName);

clear originObj;

tic;

**6. References**

1. Wan, K. X., Vidavsky, I. & Gross, M. L. Comparing similar spectra: from similarity index to spectral contrast angle. *J. Am. Soc. Mass Spectrom.* **13,** 85–88 (2002).
2. Zhang, Z. Prediction of low-energy collision-induced dissociation spectra of peptides. *Anal. Chem.* **76,** 3908-3922 (2004).
